# β-Xylosidase Overexpression Alters Pectin and Cellulose Distribution and Modulates Blast Disease Resistance in Rice

**DOI:** 10.3390/plants15060934

**Published:** 2026-03-18

**Authors:** Takashi Ohara, Taichi Watanabe, Ryuya Bamba, Atsuko Nakamura, Hiroaki Iwai

**Affiliations:** 1 Institute of Life and Environmental Sciences, University of Tsukuba, Tsukuba 305-8572, Japan; 2 Department of Biology, School of Biological Sciences, Tokai University, Sapporo 005-8601, Japan

**Keywords:** β-xylosidase, cell wall architecture, *Magnaporthe oryzae*, rice blast

## Abstract

Plant cell walls provide structural integrity and defense against biotic and abiotic stresses. In rice (*Oryza sativa*), xylan is the major hemicellulose, and β-xylosidase hydrolyzes xylan by removing xylose residues from non-reducing ends. We analyzed a transgenic rice line (*OsXylGH3-1-FOX*) that constitutively overexpresses a GH3-family β-xylosidase (Os03g0749100) under the maize ubiquitin promoter. Following inoculation with *M. oryzae*, *OsXylGH3-1-FOX* leaves exhibited increased lesion numbers and disease indices, indicating reduced resistance, whereas leaf sheaths showed fewer fungal penetrations, suggesting enhanced resistance. To investigate these organ-specific responses, we quantified cell wall components. In leaves, xylose and arabinose decreased by ~33%, and galacturonic acid (pectin) by ~50%. In leaf sheaths, xylose and arabinose were unchanged, while galacturonic acid and cellulose increased by ~50% and ~70%, respectively. Histochemical staining confirmed reduced pectin in leaves and stronger, organized cellulose and pectin in leaf sheaths. These findings suggest that decreased pectin weakens cell adhesion, facilitating pathogen ingress in leaves, whereas increased pectin and cellulose reinforce wall integrity in leaf sheaths. Thus, pectin and cellulose abundance strongly correlate with organ-specific blast resistance, while hemicellulose plays a secondary role.

## 1. Introduction

Plant cell walls are defining structures that maintain organ integrity, determine cell shape, guide differentiation and reproduction, and mediate a broad range of physiological functions [1]. As the outermost boundary of plant cells, the cell wall is the initial line of defense against biotic and abiotic stresses, such as herbivory, pathogen attack, oxidative stress, heat, intense light, salinity, and drought [2]. The primary cell wall is composed of three interwoven polysaccharide classes: cellulose, hemicelluloses, and pectins. These polysaccharides form a complex, cross-linked matrix that confers recalcitrance to saccharification and stress tolerance [3,4]. Cellulose, a β-1,4-glucan polymer, assembles into microfibrils through extensive hydrogen bonding, which imparts rigidity and load-bearing strength. Hemicelluloses (e.g., xylans, mixed-linkage glucans, and xyloglucans) associate with cellulose and lignin to stabilize the wall [5,6,7]. Pectins, which are rich in galacturonic acid (GalA), include homogalacturonan (HGA), rhamnogalacturonan I (RGI), rhamnogalacturonan II (RGII), and xylogalacturonan (XGA). The structures and substitutions of these pectins underlie wall plasticity and cell adhesion [1,8,9]. Pectin abundance varies among lineages. It is typically ~20–35% of the primary wall in dicots, but only ~5% in commelinid monocots, such as rice [10]. While cellulose composition is broadly conserved across land plants, hemicellulose composition varies by lineage: xyloglucan predominates in dicots, whereas xylan predominates in commelinid monocots (grasses) [11].

The major hemicelluloses in rice are arabinoxylans and mixed-linkage (1,3;1,4)-β-glucans. Arabinoxylans have a β-1,4-xylosyl backbone that is substituted with α-L-arabinofuranosyl side chains at O-2 and/or O-3 [12]. Xylan depolymerization occurs through the action of endo-β-1,4-xylanases (xylanases), which generate xylooligosaccharides (e.g., xylobiose), and exo-acting β-D-xylosidases (xylosidases), which remove successive xylosyl residues from the non-reducing ends of xylooligosaccharides [13,14]. Xylosidases are found in the glycoside hydrolase families GH3 and GH31; GH31 xylosidases are relatively well studied, while GH3 xylosidases are less characterized [15]. The rice gene Os03g0749100 encodes a GH3 family hydrolase with a predicted signal peptide and potential for secretion. Although GH3 enzymes are annotated with β-glucosidase activity (EC 3.2.1.21), they often exhibit broader substrate specificities, including β-xylosidase activity (EC 3.2.1.37). This implicates GH3 enzymes in xylan turnover and wall remodeling. Previous studies have shown that certain rice GH3 xylosidases, such as OsXyl1, can hydrolyze xylooligosaccharides (DP 2–6) and perform transglycosylation. This suggests that they play a role in cell wall recycling and have potential biocatalytic applications [15,16,17]. Elucidating the biochemical properties and physiological functions of Os03g0749100 could shed light on carbohydrate turnover and wall dynamics in rice.

Rice blast, caused by the fungus *Magnaporthe oryzae*, is the most destructive rice disease worldwide. After adhesion, *M. oryzae* forms an appressorium, accumulates glycerol, and generates extreme turgor pressure (up to ~8 MPa) to breach the host cell wall mechanically [18,19]. Leaf lesion severity is commonly assessed across standardized categories ranging from numerous brown specks (mild) to expanding whitish lesions (severe) [20]. To investigate how xylan catabolism influences wall architecture and disease resistance, we generated an Os03g0749100 FOX overexpression line under the maize ubiquitin-1 promoter [18,21]. Our results demonstrate that constitutive xylosidase overexpression produces organ-specific changes in wall composition and blast resistance, including decreased resistance in leaves and increased resistance in leaf sheaths. We also present biochemical and histochemical evidence that links pectin and cellulose content to these divergent outcomes.

## 2. Results

### 2.1. FOX Overexpression of a GH3 Xylosidase (Os03g0749100)

**Figure 1:**
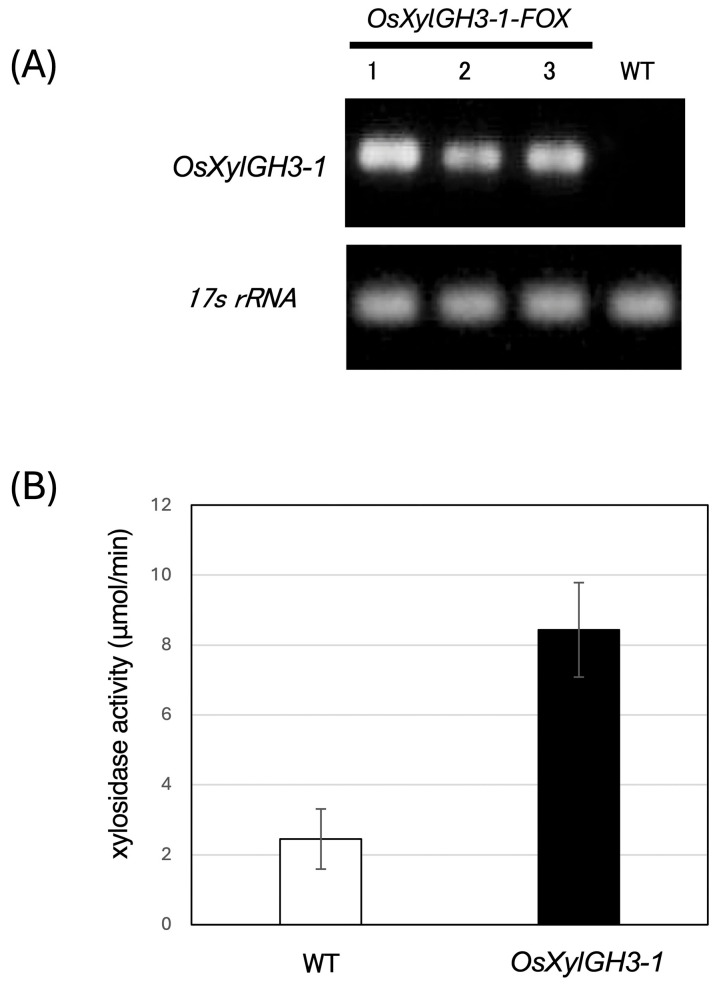
Characteristics of *OsXylGH3-1-FOX*. (**A**) RT-PCR analysis of transcripts from wild-type (WT) and OsXylGH3-1-FOX lines. The OsXylGH3-1-FOX lines exhibited higher expression levels than the WT. These analyses were performed at least three times with similar results. (**B**) β-xylosidase activities in *OsXylGH3-1-FOX* lines. Error bars show the SD (*n* = 3).

We screened ~14,500 rice FOX lines using the FOX hunting system by using expression libraries for full-length cDNAs (fl-cDNAs) from rice at a maximum of 28,000 fl-cDNA clones in total, individually overexpressed the fl-cDNAs in rice driven by the maize ubiquitin-1 gene promoter [22], and identified lines overexpressing the GH3-family xylosidase Os03g0749100. We refer to this line as *OsXylGH3-1-FOX*. Os03g0749100 transcript levels were elevated in *OsXylGH3-1-FOX* relative to wild type (WT) (Figure 1A). Consequently, xylosidase activity increased by approximately 3.4-fold in independent *OsXylGH3-1-FOX* line (line1, Figure 1A) compared to WT (Figure 1B). Under non-infected growth conditions, *OsXylGH3-1-FOX* plants did not show visible morphological or developmental abnormalities compared with WT Nipponbare (plant height, tiller number, leaf morphology, biomass).

### 2.2. Divergent Blast Resistance Responses in Leaves and Leaf Sheaths

**Figure 2:**
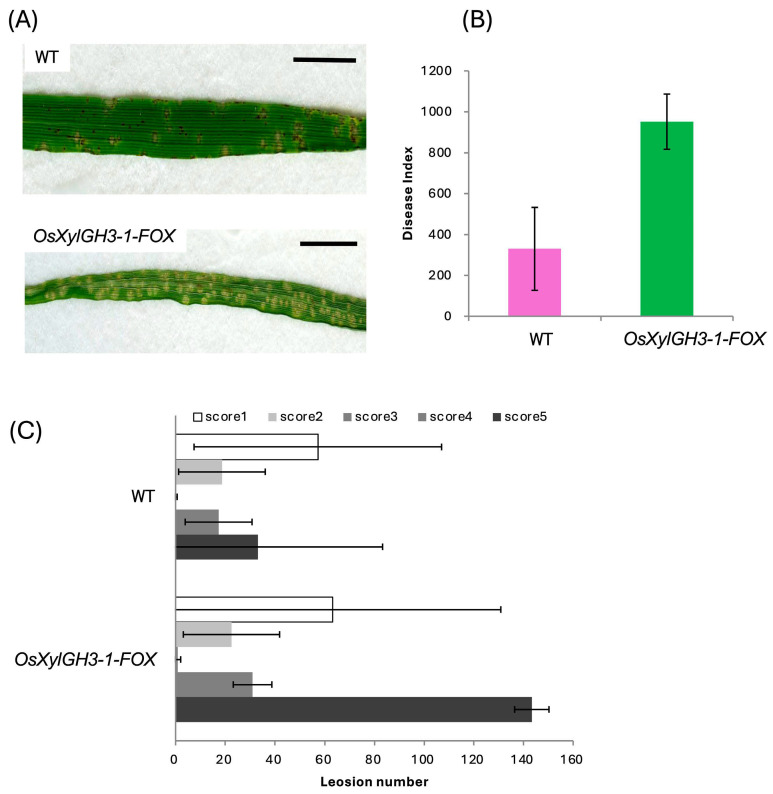
Disease Resistance of WT and *OsXylGH3-1-FOX* against Rice Blast Fungus in Leaf Blade. (**A**) Photographs of leaf blades inoculated with *Magnaporthe oryzae* conidia. Scale bars represent 10 mm. (**B**) Disease index of the WT and *OsXylGH3-1-FOX*. (**C**) Number of lesions with scores of 1–5 corresponding to necrotic spots. Error bars indicate the standard deviation (SD).

**Figure 3:**
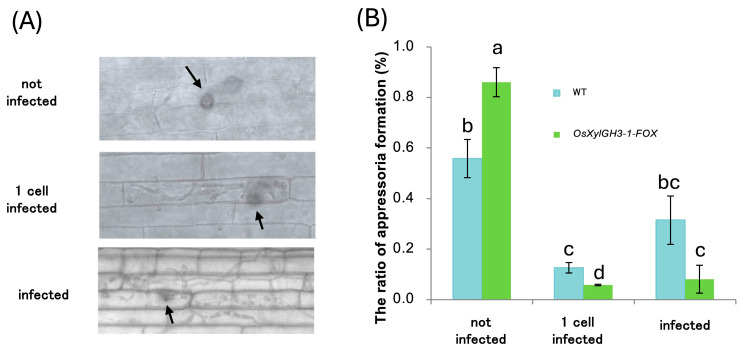
Disease Resistance of WT and *OsXylGH3-1-FOX* against Rice Blast Fungus in Leaf Sheaths. (**A**) Representative appressorium that infected one or more cells (‘infected’), one cell (‘one-cell infected’), or no cells (‘non-infected’) of the leaf sheath. Micrographs shown at standardized magnification; crystal violet staining enhances visualization of fungal structures; arrows indicate representative appressoria. (**B**) The numbers of infected, one-cell-infected, and non-infected appressoria were counted and the ratio of each was calculated relative to the total number of appressoria counted. Data are shown as mean ± SD; distinct letters denote significant differences (ANOVA/Tukey’s HSD, *p* < 0.05).

Two weeks after sowing, the fourth leaves were spray-inoculated with *M. oryzae*. *OsXylGH3-1-FOX* plants exhibited significantly higher numbers of score-5 lesions (severe whitish lesions) at 3, 5, and 7 days post-inoculation (dpi) (Figure 2A). This resulted in a disease index that was >2-fold higher than that of WT (Figure 2B). Photographs illustrate the shift toward severe lesions in *OsXylGH3-1-FOX* (Figure 2C), as opposed to a uniform distribution of lesions ranging from mild brown to severe white in WT (Figure 2A,C). For leaf sheath assays, sheaths were excised at two weeks, injected with a 1.0 × 10^5^ spores/mL suspension, fixed, and stained after 48 h. Relative to WT, *OsXylGH3-1-FOX* leaf sheaths displayed more unpenetrated appressoria and fewer single-cell penetrations and multicell expansions (Figure 3A,B), indicating enhanced resistance at the entry stage. For each replicate, 100–150 appressoria were evaluated per sheath.

### 2.3. Monosaccharide Compositions of Hemicellulose/Pectin-Enriched Fractions

**Figure 4:**
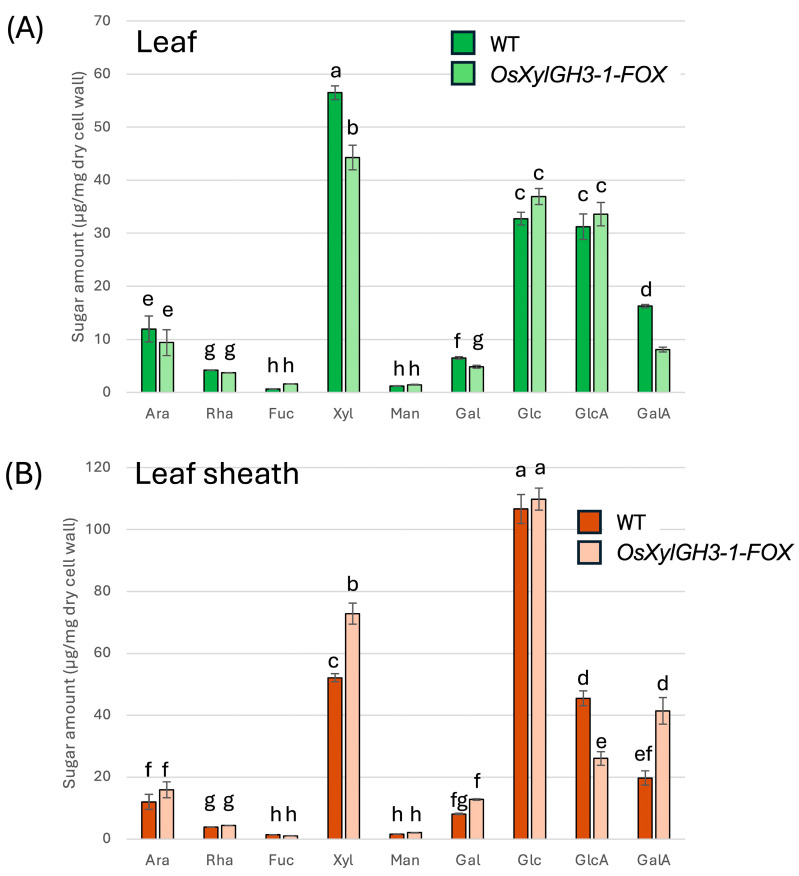
Cell Wall Component Analysis of Hemicellulose/Pectin Fractions in WT and *OsXylGH3-1-FOX* Plants. (**A**) Analysis of sugars in the trifluoroacetic acid (TFA)-soluble fraction of leaf blades in WT and *OsXylGH3-1-FOX* plants. (**B**) Analysis of sugar amounts in the TFA-soluble fraction of WT and *OsXylGH3-1-FOX* leaf sheaths. Data represent the mean of four independent biological replicates ± standard deviation (SD). Distinct letters denote significant differences (ANOVA/Tukey’s HSD, *p* < 0.05).

TFA-soluble fractions enriched in hemicellulose and pectin were analyzed by gas chromatography (GC). In leaves, *OsXylGH3-1-FOX* exhibited ~33% lower levels of xylose and arabinose (xylan signature sugars) and ~50% lower levels of galacturonic acid (GalA) (pectin signature) than WT (Figure 4A). In leaf sheaths, xylose and arabinose levels were similar to WT levels, while GalA levels increased by ~50% in *OsXylGH3-1-FOX* (Figure 4B). Statistical differences were assessed by one-way ANOVA followed by Tukey’s HSD; distinct letters indicate significant differences (*p* < 0.05).

### 2.4. Cellulose-Associated Sugar Quantification in TFA-Insoluble Fractions

**Figure 5:**
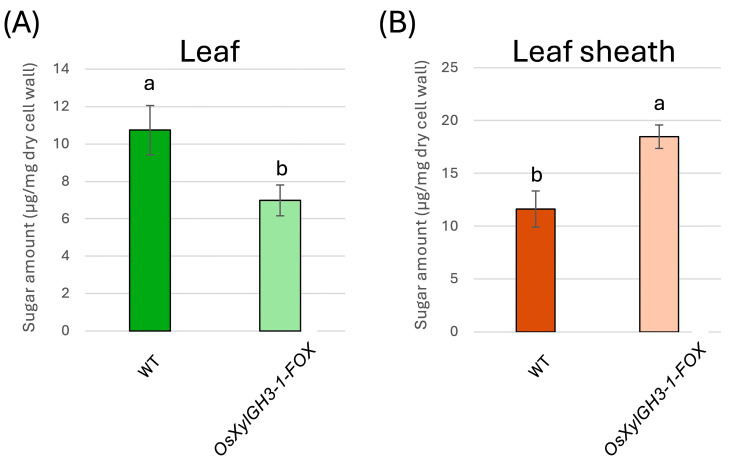
Cell Wall Component Analysis of Cellulosic Fractions in WT and *OsXylGH3-1-FOX*. (**A**) Analysis of the amount of sugars in the TFA-insoluble fraction of the leaf blade in WT and *OsXylGH3-1-FOX.* (**B**) Analysis of the amount of sugars in the TFA-insoluble fraction of WT and *OsXylGH3-1-FOX* leaf sheaths. The data represent the mean of independent biological replicates ± standard deviation (SD) for the fourth leaf (*n* = 4). Distinct letters denote significant differences (ANOVA/Tukey’s HSD, *p* < 0.05).

The neutral sugars, primarily glucose derived from cellulose hydrolysates as determined by the anthrone assay, were quantified in the TFA-insoluble fraction. Cellulose content of the leaf blade was quantified from the TFA-insoluble fraction, as shown in Figure 5A. The fourth leaf of *OsXylGH3-1-FOX* contained approximately 35% less neutral sugar than the wild type (Figure 5A), suggesting reduced cellulose levels in *OsXylGH3-1-FOX* leaves. However, the leaf sheaths of *OsXylGH3-1-FOX* had about 70% more neutral sugars than the wild type (Figure 5B), suggesting increased cellulose abundance in the *OsXylGH3-1-FOX* sheaths. Statistical differences were assessed by one-way ANOVA followed by Tukey’s HSD; distinct letters indicate significant differences (*p* < 0.05).

### 2.5. Pectin and Cellulose Staining Patterns

**Figure 6:**
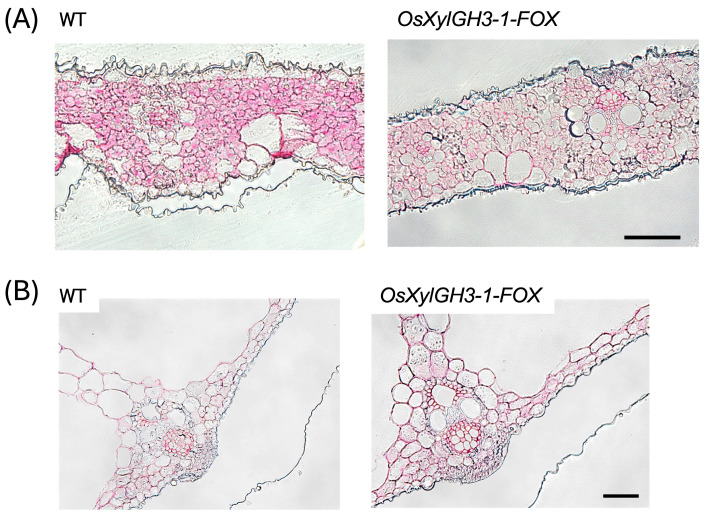
Pectin Staining of the Leaf and Leaf Sheath of WT and *OsXylGH3-1-FOX*. Pectin staining with ruthenium red was observed under bright-field illumination. (**A**) Section of leaf and (**B**) section of leaf sheath of WT and *OsXylGH3-1-FOX*. The bars represent 500 µm in (**A**) and 50 µm in (**B**). All experiments were performed at least twice with similar results.

**Figure 7:**
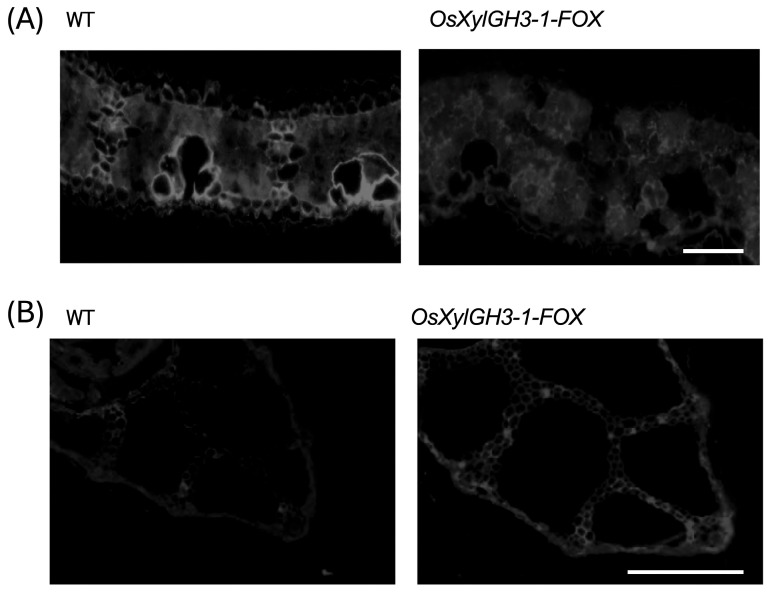
Cellulose staining of the leaf and leaf sheath of WT and *OsXylGH3-1-FOX*. Sections stained with Calcofluor White for cellulose were observed under UV illumination. (**A**) Section of leaf and (**B**) section of leaf sheath of WT and *OsXylGH3-1-FOX*. The bars represent 500 µm in (**A**) and 50 µm in (**B**). All experiments were performed at least twice with similar results.

Ruthenium red staining revealed lower pectin staining strength in *OsXylGH3-1-FOX* leaves compared to the WT (Figure 6A). Conversely, pectin staining strength was higher in *OsXylGH3-1-FOX* leaf sheaths than in the wild type (WT) (Figure 6B), which matches the biochemical increases in GalA. Leaf sections stained with toluidine blue revealed that cells in WT leaves were tightly aligned. In contrast, cells in *OsXylGH3-1-FOX* leaves were less aligned, and intercellular spaces were observed in some areas (Figure S1). Calcofluor white staining revealed localized regions of high cellulose in WT leaves (e.g., motor cells), while *OsXylGH3-1-FOX* leaves exhibited more uniform cellulose distribution (Figure 7A). In leaf sheaths, the staining strength of the cellulose staining was higher in the *OsXylGH3-1-FOX* leaves than in the WT leaves (Figure 7B). There were regularly localized regions of high cellulose in the *OsXylGH3-1-FOX* leaves.

To directly assess wall mechanics, we measured the mechanical strength of leaf blades. *OsXylGH3-1-FOX* leaves exhibited reduced breaking force compared with WT (Figure S2), consistent with compositional and staining data indicating compromised wall rigidity.

Calcofluor white staining revealed localized regions of high cellulose in WT leaves (e.g., motor cells), while *OsXylGH3-1-FOX* leaves exhibited more uniform cellulose distribution (Figure 7A). In leaf sheaths, the staining strength of the cellulose staining was higher in the *OsXylGH3-1-FOX* leaves than in the WT leaves (Figure 7B). There were regularly localized regions of high cellulose in the *OsXylGH3-1-FOX* leaves.

## 3. Discussion

Leaves of *OsXylGH3-1-FOX* exhibited significantly lower pectin content and weaker pectin staining than WT leaves (Figure 4A and Figure 6A). These leaves also showed formation of intercellular spaces (Figure S1). Both features closely resemble phenotypes reported for *OsPG2-FOX* rice lines, in which overexpression of a pectin degrading polygalacturonase reduces pectin, weakens cell adhesion, enlarges intercellular gaps, and facilitates *M. oryzae* ingress under conducive conditions [19]. Mechanistically, the pectin rich middle lamella is the principal mediator of plant cell–cell adhesion; perturbation of pectin abundance or cross linking compromises tissue integrity and increases wall porosity, thereby lowering the physical barrier to invasion [23,24]. These biophysical changes are significant because *M. oryzae* appressoria generate high turgor pressure (~8 MPa) and a rigid penetration peg, which depends on mechanical force to breach the cuticle and primary cell wall. Compared with the wild type, the leaves of *OsXylGH3-1-FOX* exhibited reduced cellulose content, which is a major determinant of cell wall strength, and diminished cellulose staining (Figure 5A and Figure 7A). Consequently, a decrease in wall strength or structural continuity can lower the threshold required for fungal [22,25]. Consistent with this interpretation, direct mechanical measurements showed reduced leaf mechanical strength in *OsXylGH3-1-FOX* relative to WT (Figure S2). We did not directly measure the mechanical properties of leaf sheaths, which is a limitation of the present work. A further limitation of this study is that the primary phenotypic analyses are based on a single FOX overexpression line. While FOX lines originate from independent full-length cDNA insertions, a more definitive understanding of the role of *OsXylGH3-1* would require additional independent overexpression lines or loss-of-function mutants. Although we did not directly test Os03g0749100 activity on native rice xylan in vitro, several lines of evidence support its functional annotation as a GH3 β-xylosidase: (i) conserved GH3 catalytic domains and active-site features characteristic of β-xylosidases [11]; (ii) close homology to rice OsXyl1 with verified β-xylosidase activity on xylooligosaccharides (DP 2–6) [17]; and (iii) in vivo reductions in arabinoxylan signature sugars (xylose and arabinose) specifically in leaves (Figure 4A), consistent with xylan turnover in planta. In addition to differences in wall composition, leaf blades and leaf sheaths differ in development and anatomy. Leaf blades are photosynthetically active tissues that are highly expanded with thinner supporting structures and larger intercellular spaces. Leaf sheaths, on the other hand, consist of more compact, mechanically reinforced tissues that support the culm. These architectural and functional distinctions likely make blades more sensitive to reductions in pectin and cellulose while enabling sheaths to strengthen their wall matrices in response to elevated levels of these substances, thereby contributing to the divergent resistance phenotypes observed.

The observed decreases in xylose and arabinose in *OsXylGH3-1-FOX* leaves are consistent with β xylosidase overactivity on xylan/arabinoxylan side chains. A rice GH3 β-xylosidase (OsXyl1) efficiently hydrolyzes xylooligosaccharides, supporting a role in xylan turnover in planta [17]. Although a direct causal chain from xylan depletion to pectin loss is not yet defined in rice, covalent xylan–pectin–AGP linkages (the APAP1 proteoglycan) have been demonstrated in Arabidopsis, showing that hemicellulose and pectin domains can be structurally integrated; selective depletion of one domain can alter the extractability and organization of the other [21]. Together, these data support the inference that decreased pectin—and possibly secondary consequences of altered xylan–pectin connectivity—compromise adhesion and plausibly underlie reduced leaf resistance in *OsXylGH3-1-FOX*. In this study, β-xylosidase activity was quantified only in leaf extracts; we did not independently measure activity in leaf blades versus leaf sheaths, nor did we determine subcellular localization (apoplast vs. intracellular). Thus, tissue-specific differences in enzyme abundance, activation, or localization could contribute to the contrasting organ-level phenotypes. Future work employing tissue-resolved enzyme assays and localization analyses will help disentangle differential enzyme action from downstream remodeling.

In contrast, *OsXylGH3-1-FOX* leaf sheaths showed higher pectin content and staining (Figure 4B and Figure 6B), indicating improved adhesion. Moreover, cellulose content and staining strength were markedly increased (Figure 5B and Figure 7B). Because cellulose is the principal load-bearing component that confers mechanical rigidity, cellulose-rich walls strengthen the physical barrier at the penetration stage [26]. A stiffer, more continuous sheath wall should resist the penetration peg emergence that appressoria require to invade host tissue [25]. Consistent with this, several studies show that host cell wall strengthening—including lignification of epidermal mechanical tissues—can impede early penetration by blast fungus [27], and broader reviews of cell wall–associated immunity highlight coordinated remodeling of cellulose, pectin, and lignin as a front line defense [28,29].

Although levels of hemicellulose signature sugars decreased in *OsXylGH3-1-FOX* leaves, no significant change was detected in leaf sheaths (Figure 4). Relative to pectin (adhesion/plasticity) and cellulose (rigidity), hemicellulose appears secondary for determining the organ-specific resistance outcomes observed here. That said, recent biophysical work shows glucuronoarabinoxylan (GX) binds cellulose surfaces in a specific two-fold screw conformation and is critical for proper bundling and alignment of cellulose microfibrils in secondary walls; perturbing xylan can disrupt cellulose network architecture and mechanical performance [30]. Beyond structure, xylan-derived arabinoxylan oligosaccharides (AXOS) act as damage-associated molecular patterns (DAMPs) that trigger immune responses, and monocot xylanase inhibitors (TAXI/XIP/TLXI) contribute to wall-based immunity by inhibiting pathogen CWDEs [31,32]. Thus, hemicellulose can modulate resistance indirectly—via cellulose organization and immune signaling—even if its bulk content is not the primary determinant of the leaf vs. sheath phenotypes reported here.

A further limitation is that we did not assess immune signaling outputs (e.g., PR gene expression, ROS accumulation, or callose deposition) in leaves and sheaths before/after infection. Because cell wall remodeling interfaces with cell wall integrity sensing and downstream defense activation, future work should determine whether altered wall architecture in *OsXylGH3-1-FOX* modulates these pathways in addition to mechanical resistance.

Compared with *OsPG2-FOX*, which directly degrades homogalacturonan and recapitulates reduced adhesion and increased leaf susceptibility [19], *OsXylGH3-1-FOX* likely acts via xylan–pectin cross-domain interactions that secondarily impact pectin abundance/extractability. Notably, the organ-specific divergence we report here (leaf susceptibility vs. sheath resistance) has not, to our knowledge, been described for *OsPG2-FOX* or other wall-modifying lines, underscoring tissue-dependent consequences of β-xylosidase-mediated remodeling.

Constitutive overexpression of a GH3 β-xylosidase in rice produces organ-specific cell wall remodeling and divergent blast resistance phenotypes. In leaves, reduced pectin compromises adhesion and increases susceptibility. In leaf sheaths, increased pectin and cellulose strengthen wall integrity and improve resistance to penetration. These findings support a positive correlation between pectin/cellulose abundance and blast resistance, with hemicellulose contributing secondary effects. Future work should assess defense gene expression, lignification, and the biophysical properties of cell walls to further elucidate how xylosidase-driven remodeling shapes organ-specific immunity. Together with new mechanical data for leaves (Figure S2) and explicit limitations noted above, these results provide an integrated view of how xylosidase-driven remodeling shapes organ-specific immunity and mechanics.

## 4. Materials and Methods

### 4.1. Plant Material and Growth Conditions

The rice plants used were of the wild type (*Oryza sativa* cv. Nipponbare) and of the FOX line BP164, which carries an overexpression construct for OsXylGH3-1 (Os03g0749100). The plants were grown in a growth chamber at 28 °C with 115 µmol s^–1^ m^–2^ illumination. The presence of the constructs in the genomic DNA was confirmed by PCR using third-generation plants. Transgenic lines were selected on hygromycin-containing agar and tested for heritable expression patterns and altered sugar traits.

To analyze gain-of-function phenotypes, the FOX hunting system was developed using full-length cDNA (fl-cDNA) expression libraries from rice containing 28,000 fl-cDNA clones. They overexpressed the fl-cDNAs in rice under the control of the maize ubiquitin-1 promoter [18]. Among ~14,500 FOX rice lines, one overexpressed fl-cDNAs for xylosidase (accession no. AK102309; Rap-ID Os03g0749100) in GH family 3 and was named *OsXylGH3-1-FOX*. All FOX lines were compared to the WT Nipponbare background following standardized FOX-hunting procedures [18].

### 4.2. RNA Extraction and Gene Expression Analysis

The plant material was frozen in liquid nitrogen and ground using a TissueLyser II (Qiagen, Hilden, Germany) [19]. Total RNA was extracted using an RNeasy Plant Mini Kit (Qiagen, Hilden, Germany) and DNase I (Roche, Basel, Switzerland) following the manufacturer’s instructions.

cDNA was synthesized using ReverTra Ace^®^ (Toyobo, Tokyo, Japan) according to the manufacturer’s specifications. For the *OsXylGH3-1-FOX* line, transcripts were quantified using the primers OsXylGH3-1-forward (5′-TGCCACGTACTTGGTTCAA-3′) and OsXylGH3-1-reverse (5′-TAACCCCTAACTCCGGCTC-3′). The 17S rRNA transcript was used as an endogenous control because the small subunit rRNA in rice is a 17S molecule, as established by the complete sequencing of rice nuclear rDNA [33]. Primer specificity was confirmed by NCBI BLAST online tool against the *Oryza sativa* genome, and the amplicon size was verified by gel electrophoresis to match the expected product. We removed duplicated sentences and streamlined the paragraph for clarity.

### 4.3. β-Xylosidase Assay

The rice leaf blades were placed in a crushing tube, frozen in liquid nitrogen, and crushed using a TissueLyser (Qiagen). Then, one volume of extraction buffer (50 mM Tris-HCl, 3 mM EDTA, 1 M NaCl, and 10% glycerol [*v*/*v*]) was added. The sample was gently mixed and allowed to stand at 4 °C for two hours. Next, the sample was centrifuged at 15,000 rpm and 4 °C for 15 min. Finally, the supernatant was collected and used as the crude enzyme solution.

Enzymes were extracted in a buffer made with sodium acetate (20 mM) and NaCl (1 M). The pH of the buffer was 5.0. Each tissue was ground in liquid nitrogen, extracted in sodium acetate buffer, and then centrifuged at 15,000× *g* for 30 min at 4 °C. The hydrolytic activities of the cell wall proteins were determined. The cell wall proteins were extracted from each tissue. They were determined toward synthetic substrates. A reaction mixture was used. It consisted of the cell wall proteins, 25 mM acetate buffer (pH 5.0), and 1 mM PNP-glycoside substrate. The reaction was terminated by the addition of 200 mM sodium carbonate (800 µL) after being incubated at 37 °C for 2 h. The color change was measured at 420 nm. One unit of enzyme activity liberates 1 µmol of p-nitrophenol per minute.

### 4.4. Extraction and Analysis of Cell Wall Polysaccharides

Cell wall extraction and analysis were conducted according to Sumiyoshi et al., with slight modifications [9]. Full, mature leaves were frozen in liquid nitrogen and ground using a TissueLyser II (Qiagen) at 30 Hz for two minutes. The resulting powder was washed with 80% ethanol. After centrifugation for five minutes at 15,000 g, the supernatant was decanted and the pellet was treated with methanol and chloroform (MC; 1:1, *v*/*v*); acetone; and phenol, acetic acid, and water (PAW; 2:1:1, *v*/*v*). After amylase treatment to remove starch, the samples were air-dried, and the resulting alcohol-insoluble residue (AIR) was used as cell wall material. The AIR (2 mg) was hydrolyzed in 2 M trifluoroacetic acid (TFA), and the resulting pellets were hydrolyzed in 72% H_2_SO_4_. The resulting fractions were treated with methanol and hydrogen chloride. The methyl glycosides were then converted into trimethylsilyl derivatives and analyzed by gas–liquid chromatography (GC-2010; Shimadzu, Kyoto, Japan). The hexose and uronic acid contents were determined by the anthrone and meta-hydroxybiphenyl methods, respectively.

### 4.5. Microscopy

The following procedure is based on the method described by Hasegawa et al. [34]. A fully expanded leaf was fixed for 2 h in 4% paraformaldehyde, 0.25% glutaraldehyde, and 0.05 M phosphate buffer at room temperature. Transverse 3 µm sections were cut using a Leica RM2145 microtome (Leica Microsystems, Wetzlar, Germany). The sections were stained for 5 min with 0.02% (*w*/*v*) ruthenium red in DW and visualized by light microscopy. For cellulose staining, sections were treated with 0.01% (*w*/*v*) calcofluor white (Fluorescent Brightener 28, FUJIFILM Wako Pure Chemical Corporation, Osaka, Japan) for 1 min, washed three times with DW, and visualized by fluorescence microscopy (Leica, Wetzlar, Germany; DMRB).

### 4.6. Assay of Disease Resistance

For spray inoculation, intact rice seedlings (fourth leaf stage) were grown in a hydroponic culture for three weeks, as previously described [19]. The fourth leaves were excised and placed on moist filter paper in a clear plastic box. The leaves were spot-inoculated with a 10 µL suspension of *M. oryzae* (strain P91-15B; race 001; 86–137) conidia (1 × 10^5^ or 10^6^ mL^−1^) and incubated at 25 °C in the dark for two days. Subsequently, the leaves were incubated at 25 °C under continuous white light for five days. Disease symptoms were recorded at 3, 5, and 7 dpi. Lesion size was measured and the disease index was calculated according to Toyomasu et al. [35]. For the Blast Resistance Test by Leaf Sheath Inoculation, rice plants were grown in an incubator for two weeks, and cultured blast fungi were used as in the blast fungus spray experiment. The rice plants were cut approximately 1 cm from the root at the bottom and 1 cm from the leaf base at the top. The intermediate sections were used as leaf sheaths for the experiment. A spore suspension adjusted to a concentration of 1 × 10^5^ spores/mL was injected into the leaf sheaths using a syringe. Cardboard was placed in a square Petri dish and moistened with deionized water (DW) sufficient to maintain humidity for approximately one day. A toothpick was placed on top of the cardboard to keep the leaf sheaths from touching it directly. The injected leaf sheaths were then placed on top of the toothpick. The Petri dish was covered with a lid and sealed with mending tape. It was then left in the dark at 28 °C for 48 h. The mycelia that had penetrated the cells were stained with crystal violet and observed. For each replicate, 100–150 appressoria were evaluated. Magnification and field of view were standardized across samples. Penetration categories were scored as defined in Figure 3A.

### 4.7. Mechanical Strength Measurements

The mechanical properties of leaves were measured following the method described by Ohara et al. [19]. Mature leaves were cut approximately 12 cm from the tip, mounted with a 2 cm interval between both ends, and positioned so that the midpoint of the sample was placed over the sensor. Breaking force (N/mm) and strain (%) were measured using a force tester (MCT-1150, A&D COMPANY, Ltd., Tokyo, Japan).

### 4.8. Statistical Analysis

The data were expressed as the mean values ± standard deviations (SD) taken from 4–9 biological independent experiments. The experimental data of the samples were statistically analyzed through one-way analysis of variance (ANOVA) with Tukey’s post hoc test using Statistica 13.1 software (StatSoft, Inc., Tulsa, OK, USA). The results with *p*-value ≤ 0.05 and *p*-value ≤ 0.01 were considered statistically significant. Figure 3, Figure 4 and Figure 5 include statistical annotations; distinct letters denote significant differences (*p* < 0.05) based on ANOVA/Tukey’s HSD. For clarity, the number of biological replicates is summarized as follows: RT-PCR (*n* = 3), enzyme assays (*n* = 3), GC sugar analysis (TFA-soluble and TFA-insoluble; *n* = 4), leaf blade disease assays (*n* = 5–9 independent leaves), leaf sheath penetration assays (*n* = 4 sheaths; 100–150 appressoria/replicate), and histochemical staining (≥2 independent experiments with similar results). All comparisons are against WT Nipponbare. All statistical analyses were conducted within a hypothesis-driven framework in which predefined comparisons between the WT and the FOX line were evaluated using one-way ANOVA followed by Tukey’s HSD.

## Data Availability

The original contributions presented in this study are included in the article/Supplementary Material. Further inquiries can be directed to the corresponding author.

## Supplementary Materials

The following supporting information can be downloaded at https://www.mdpi.com/article/10.3390/plants15060934/s1, Figure S1: Cross-sections of the fourth leaves from WT and *OsXylGH3-1-FOX* observed by light microscopy. Figure S2: Mechanical properties of leaves of WT and *OsXylGH3-1-FOX.*
