# Supplementary material for: β-Xylosidase Overexpression Alters Pectin and Cellulose Distribution and Modulates Blast Disease Resistance in Rice
Source: Plants (Basel). 2026 Mar 18;15(6):934. doi: 10.3390/plants15060934 (PMC13030476; doi:10.3390/plants15060934)
Supplement: Supplementary file 1 [file plants-15-00934-s001.zip › plants-4136753-supplementary.pdf]

plants

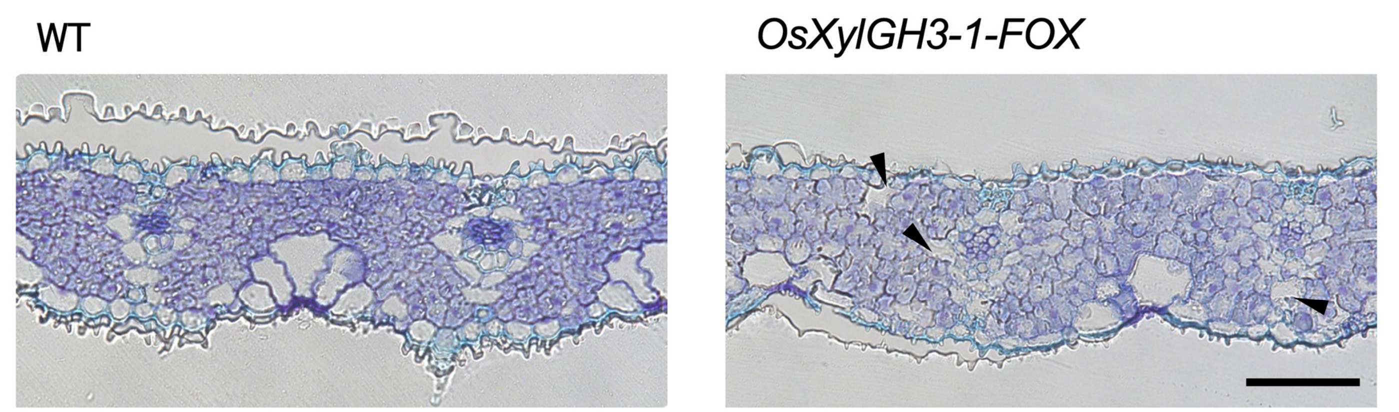

Figure S1. Cross-sections of the fourth leaves from WT and *OsXylGH3-1-FOX* observed by light microscopy. Sections were stained with toluidine blue O. Cross-sections from the WT and *OsXylGH3-1-FOX*. The intercellular spaces (arrowheads) can be observed in *OsXylGH3-1-FOX*. Scale bars represent 500 µm.

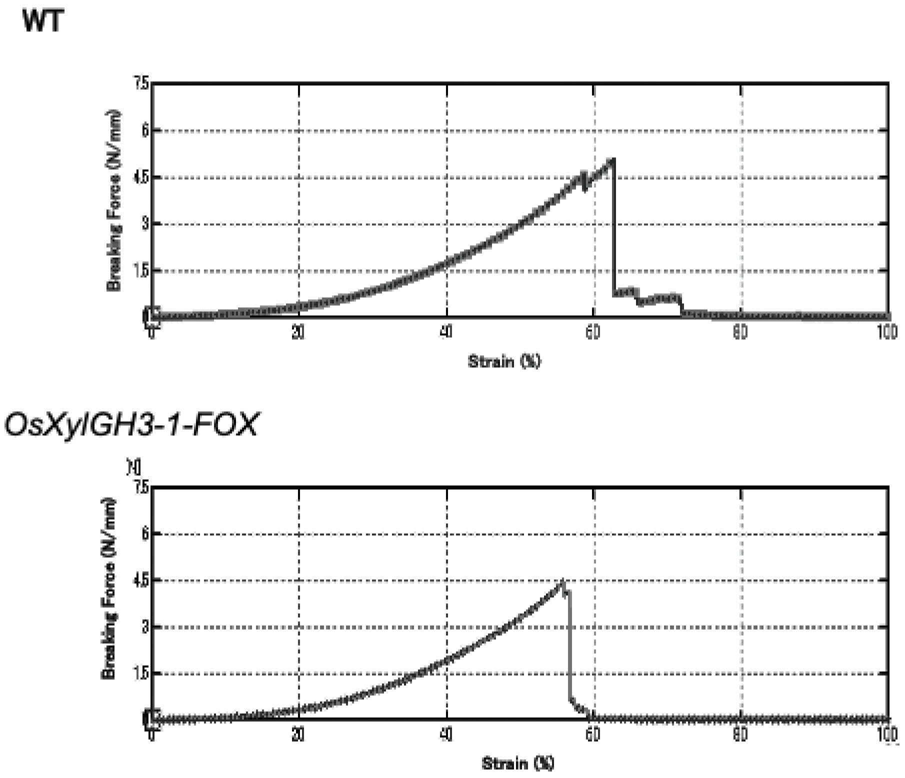

Figure S2. Mechanical properties of leaves of WT and OsXylGH3-1-FOX. Strain and breaking force when the leaf blade broke. The horizontal axis shows the extension length.
